# Cryo-EM structure and in vitro DNA packaging of a thermophilic virus with supersized T=7 capsids

**DOI:** 10.1073/pnas.1813204116

**Published:** 2019-02-08

**Authors:** Oliver W. Bayfield, Evgeny Klimuk, Dennis C. Winkler, Emma L. Hesketh, Maria Chechik, Naiqian Cheng, Eric C. Dykeman, Leonid Minakhin, Neil A. Ranson, Konstantin Severinov, Alasdair C. Steven, Alfred A. Antson

**Affiliations:** ^a^York Structural Biology Laboratory, Department of Chemistry, University of York, York YO10 5DD, United Kingdom;; ^b^Laboratory of Structural Biology Research, National Institute of Arthritis Musculoskeletal and Skin Diseases, National Institutes of Health, Bethesda, MD 20892;; ^c^Center for Life Sciences, Skolkovo Institute of Science and Technology, 143025 Skolkovo, Russia;; ^d^Institute of Molecular Genetics, Russian Academy of Sciences, 123182 Moscow, Russia;; ^e^Astbury Centre for Structural Molecular Biology, University of Leeds, Leeds LS2 9JT, United Kingdom;; ^f^Department of Mathematics, University of York, York YO10 5DD, United Kingdom;; ^g^Waksman Institute for Microbiology, Rutgers, The State University of New Jersey, Piscataway, NJ 08854

**Keywords:** virus assembly, DNA packaging, capsid, portal protein, cryo-EM

## Abstract

Double-stranded DNA viruses, including bacteriophages and herpesviruses, package their genomes into preformed capsids, using ATP-driven motors. Seeking to advance structural and mechanistic understanding, we established in vitro packaging for a thermostable bacteriophage, P23-45 of *Thermus thermophilus*. Both the unexpanded procapsid and the expanded mature capsid can package DNA in the presence of packaging ATPase over the 20 °C to 70 °C temperature range, with optimum activity at 50 °C to 65 °C. Cryo-EM reconstructions for the mature and immature capsids at 3.7-Å and 4.4-Å resolution, respectively, reveal conformational changes during capsid expansion. Capsomer interactions in the expanded capsid are reinforced by formation of intersubunit β-sheets with N-terminal segments of auxiliary protein trimers. Unexpectedly, the capsid has T=7 quasi-symmetry, despite the P23-45 genome being twice as large as those of known T=7 phages, in which the DNA is compacted to near-crystalline density. Our data explain this anomaly, showing how the canonical HK97 fold has adapted to double the volume of the capsid, while maintaining its structural integrity. Reconstructions of the procapsid and the expanded capsid defined the structure of the single vertex containing the portal protein. Together with a 1.95-Å resolution crystal structure of the portal protein and DNA packaging assays, these reconstructions indicate that capsid expansion affects the conformation of the portal protein, while still allowing DNA to be packaged. These observations suggest a mechanism by which structural events inside the capsid can be communicated to the outside.

## Significance

Understanding molecular events during virus assembly and genome packaging is important for understanding viral life cycles, and the functioning of other protein–nucleic acid machines. The model system developed for the thermophilic bacteriophage P23-45 offers advantages over other systems. Cryo-EM reconstructions reveal modifications to a canonical capsid protein fold, resulting in capsids that are abnormally large for this virus class. Structural information on the portal protein, through which the genome is packaged, demonstrates that the capsid influences the portal’s conformation. This has implications for understanding how processes inside and outside the capsid can be coordinated.

A key step in the assembly pathway of double-stranded DNA viruses, including bacteriophage and evolutionarily related herpesviruses, is the packaging of viral genomic DNA into the procapsid (1). During this process, the large terminase protein docks onto the portal protein, which is embedded in the capsid wall, and translocates DNA using free energy liberated from ATP hydrolysis. This motor can work against high internal pressure, generating forces reaching 60 pN and translocating DNA at rates reaching 100 bp/s to 2,000 bp/s (2, 3). As a result, DNA is packaged inside the virion to near-crystalline density (>500 mg/mL) (4, 5). This makes the motor a suitable tool for biotechnological applications such as gene delivery or sequencing, and a potential target for drug discovery in the case of human and animal viruses (6). The unique features of this motor also make it a useful system for studying fundamental biological processes involving DNA translocation and the coupling between ATP hydrolysis and mechanical work.

A number of mesophilic bacterial viruses have been employed to study DNA packaging in vitro (7–13). Initial studies on bacteriophage λ demonstrated that procapsids were precursors to the expanded head (14) and that purified empty capsids can be packaged with double-stranded DNA in the presence of the ATPase that drives genome packaging—the large terminase—and ATP (15). The in vitro packaging reaction enables this key stage in virus assembly to be studied under controlled conditions, mimicking events inside the host cell. ATP consumption as well as packaging rates and forces have been measured using in vitro systems established for bacteriophages ϕ29, T4, and λ (2, 16, 17). In parallel, cryo-EM 3D reconstructions have been reported for capsid–motor complexes of bacteriophages ϕ29 and T4, to resolutions of 12 Å and 34 Å, respectively (18, 19). Combined with crystal structures of individual motor components, pseudoatomic models have begun to show how the motor engages with DNA and how translocation is achieved. However, the precise molecular details for the packaging mechanism have yet to be determined. This research would benefit from the availability of a robust packaging system with greater stability.

To advance structural and mechanistic studies, we have established an in vitro DNA packaging system for the thermophilic virus P23-45. P23-45 and P74-26 belong to the genus *P23virus*, and are closely related to bacteriophage G20c, all of which infect *Thermus thermophilus* (20). Individual components of the DNA packaging motor of these viruses have been characterized biochemically and structurally (21–24). We isolated procapsids and expanded capsids of P23-45 and demonstrated DNA packaging in vitro in the presence of cognate large terminase gp85. Despite its large genome, which is twice as big as that of HK97, P23-45 utilizes a similar capsid protein fold, and forms capsids with the same T=7 quasi-symmetry as HK97 and similar phages. Cryo-EM reconstructions explain the structural basis for this anomalous size, showing how the larger capsid lattice is accomplished by modifying the canonical HK97 fold, and how the conformation of the capsid protein changes during capsid expansion. One of the differences between the procapsid and expanded capsid is the presence of trimers of an auxiliary protein on the outer surface of the expanded capsid, a property held in common with λ and TW1 (25–27). Furthermore, reconstructions of the capsid which resolve the unique portal vertex, combined with a 1.95-Å resolution crystal structure of the portal protein, allowed structural characterization of the unique portal–capsid interface, where the respective symmetries of interacting proteins do not match. These data show how the portal protein structure is affected by capsid expansion: Combined with normal mode analysis calculations, they suggest the existence of cross-talk between parts of the portal protein that are respectively inside and outside the capsid.

## Results

### Cryo-EM Icosahedral Capsid Reconstructions.

**Fig. 1.**
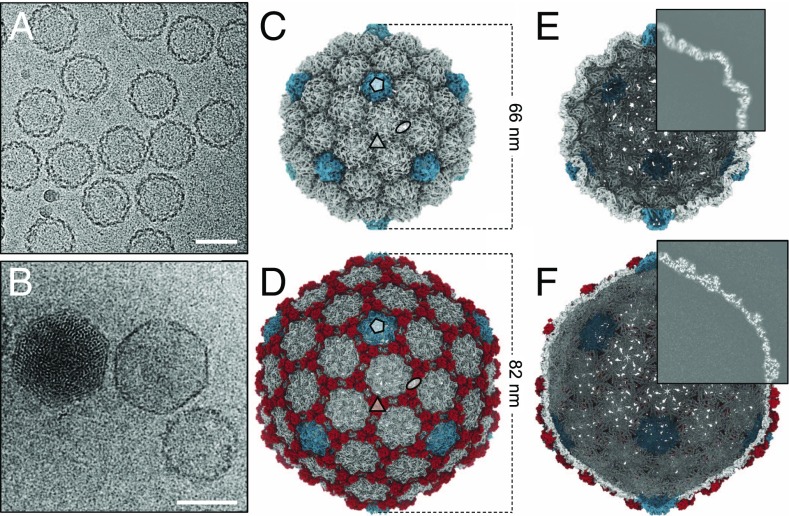
Cryo-EM analysis. Micrographs showing (*A*) procapsids and (*B*) DNA-filled capsid (left), empty expanded capsid (center), and empty procapsid (right). (Scale bars, 50 nm.) (*C* and *D*) Icosahedral 3D reconstruction of the (*C*) procapsid and (*D*) empty expanded capsid, symmetry axes indicated. Pentons are in cyan, auxiliary proteins in red. (*E* and *F*) Internal views of the (*E*) procapsid and (*F*) expanded capsid, with one-quarter of the reconstruction shown as a central slice.

**Fig. 2.**
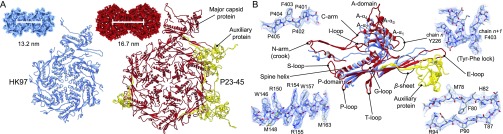
Comparison of lattice spacing in expanded capsids of P23-45 and HK97. (*A*) (*Top*) Spacing between adjacent hexons of HK97 (blue, left) and P23-45 (red, right) shown in space-filling representation. (*Bottom*) Icosahedral asymmetric unit of each phage shown as ribbon diagrams. For P23-45, the major capsid proteins are in red and auxiliary proteins are in yellow. (*B*) Overlay of the P23-45 (red) and HK97 (blue) major capsid monomers shown as ribbon diagrams. Representative segments of the atomic model of P23-45 with corresponding regions of the map. Chain *n + 1* represents the counterclockwise chain from chain *n* in a hexon when viewed from the outside of capsid.

Spherical capsids with thick serrated walls (Fig. 1*A*) were present alongside larger, faceted capsids with thinner walls (Fig. 1*B*) in lysates of P23-45–infected *T. thermophilus* cells. The smaller capsids, hereafter referred to as procapsids, were in higher abundance than the mature expanded empty capsid. Empty and DNA-filled expanded capsids appeared to be of the same size (Fig. 1*B*), with the diameter of the circumsphere at ∼82 nm, compared with ∼66 nm for the procapsid. Single-particle reconstructions imposing icosahedral symmetry (Movies S1 and S2) were calculated at 4.4-Å resolution for the procapsid and 3.7-Å resolution for the expanded capsid (Fig. 1 *C*–*F* and *SI Appendix*, Table S1). These capsids all exhibited T=7 *laevo* quasi-symmetry (28). In the case of the expanded capsid, there were interpretable regions for side chains for most amino acids, enabling atomic models to be built for both the major capsid protein gp89 (*SI Appendix*, Fig. S1) and the auxiliary protein gp88 (*SI Appendix*, Fig. S2). The model of the major capsid protein revealed the canonical HK97-like fold (29), with major insertions that result in a supersized capsid geometry with an increased lattice constant (Fig. 2). The model of the major capsid protein obtained for expanded capsids was adjusted to fit the lower-resolution cryo-EM map of the procapsid. All residues for the expanded capsid could be modeled, whereas the procapsid model contains all residues except for 23 N-terminal and 12 C-terminal amino acids, for which there is no clearly defined density, suggesting flexibility or disorder. Segments of residues 24 to 45, 62 to 76, 93 to 105, and 126 to 136 were modeled as poly-Alanine due to lack of interpretable side-chain density.

### Capsid Protein Fold and Intersubunit Interactions.

The HK97 capsid monomer can be subdivided into three domains: A domain, making up the apex of each capsomer; P domain, found at the periphery of each capsomer; and E loop, extending from the P domain. The icosahedral asymmetric unit contains seven copies of the major capsid protein (Fig. 2*A*). The expanded P23-45 capsid differs by the presence of auxiliary protein gp88 trimers, which are found at local and icosahedral threefold axes, as in phage λ (25). The larger volume of the P23-45 capsids compared with other T=7 capsids (Table 1) results from an increased spacing between capsomers within the icosahedral lattice. This spacing has been observed to be 13 nm and 14 nm in viruses with the HK97 fold, irrespective of the T number (30, 31). In the P23-45 capsids, however, capsomers are repeated at a ∼25% larger interval of ∼17 nm (Fig. 2*A*).

**Table 1.** Internal volumes of bacteriophage capsids

Phage	T-number	V_p_,* 10^4^ nm^3^	V_e_, 10^4^ nm^3^	Genome size, kb
HK97	7	3.88 (0.47)	8.25	39.7
T7	7	4.79 (0.55)	8.64	39.9
P22	7	5.25 (0.58)	9.09	41.7
λ	7	4.88 (0.51)	9.50	48.5
SIO-2	12	9.26 (0.59)	15.7	81.2
P23-45	7	8.90 (0.49)	18.2	84.2

Volume calculations for procapsids (V_p_) and expanded capsids (V_e_) were performed for the following structures: HK97 = EMD 5828, EMD 2112, PDB 1OHG; T7 = EMD 6034, EMD 6037; P22 = EMD 1824, EMD 1826; λ = EMD 1507, EMD 5012; and SIO-2 = EMD 5383, EMD 5382.

* V_p_ as a fraction of V_e_ is given in parentheses.

Analysis of the structure and comparison with the canonical HK97 fold shows that two key factors contribute to the increased capsid lattice spacing. First, the A domains of the P23-45 capsid protein are extended. Compared with HK97, the two conserved helices A-α_1_ (213 to 224) and A-α_2_ (242 to 247) (Fig. 2*B*), corresponding to residues 254 to 268 and 279 to 286 in HK97, respectively, are shifted away by ∼10 Å from the P domain in P23-45 owing to extensions to loops 202 to 212 and 318 to 321. In addition, the A domain of P23-45 has two further α-helices, A-α_3_ (249–258) and A-α_4_ (272 to 287), which protrude into the middle of the capsomer apex. Second, the P23-45 capsid protein has a longer E loop than other HK97-like viruses, extending ∼12 Å farther. This complements the increased capsomer diameter, allowing the E loop to reach far enough to make interactions with neighboring subunits.

**Fig. 3.**
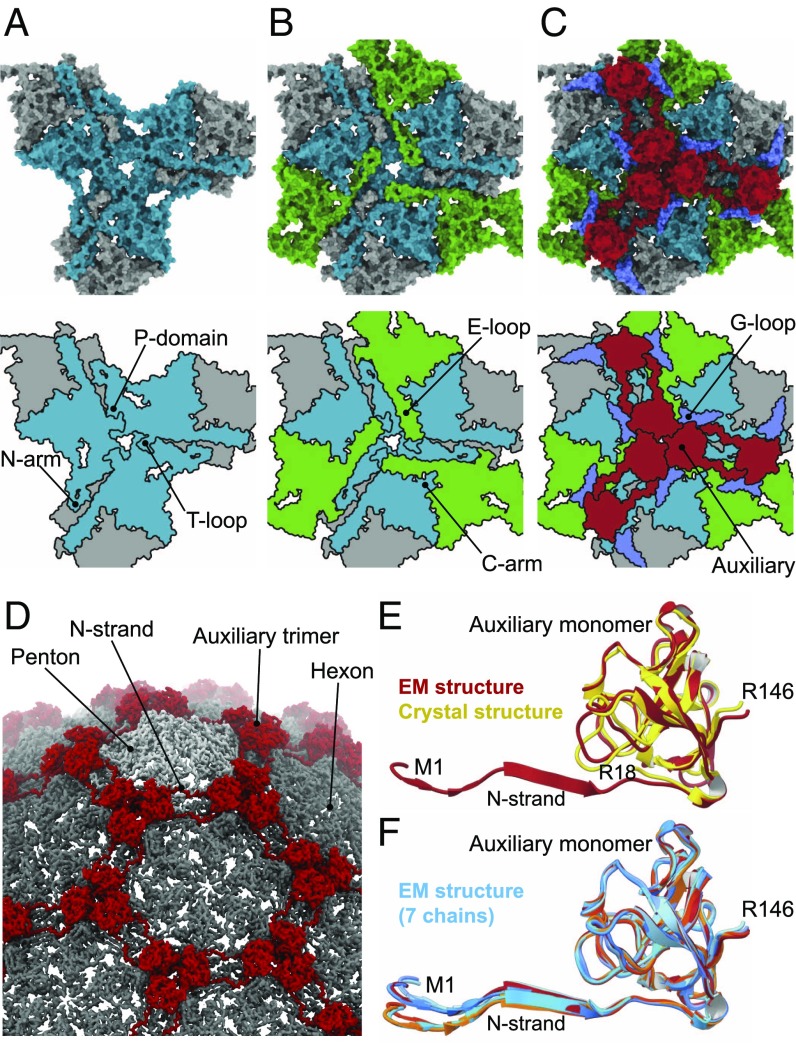
The multilayered nature of subunit interactions. (*A*) Innermost layer of the capsid wall featuring six capsid protein chains centered on a threefold symmetry axis, shown in gray and cyan. (*B*) A middle layer featuring E loops from three further subunits (green) extending toward the central axis and overlaying the P domains of subunits in the innermost layer. (*C*) Upper layer containing auxiliary protein trimers (red), and major capsid protein G loops (blue) from the internal layer. Space-filling representations in *A*–*C*, *Top* are shown along with schematic diagrams (*Bottom*). (*D*) Segment of the expanded capsid map with annotated features. (*E*) Overlay of the auxiliary protein monomer from the cryo-EM reconstruction (red, this work) and crystal structure (yellow, PDB ID code 6BL5). (*F*) Overlay of the seven auxiliary protein subunits constituting the icosahedral asymmetric unit.

**Fig. 4.**
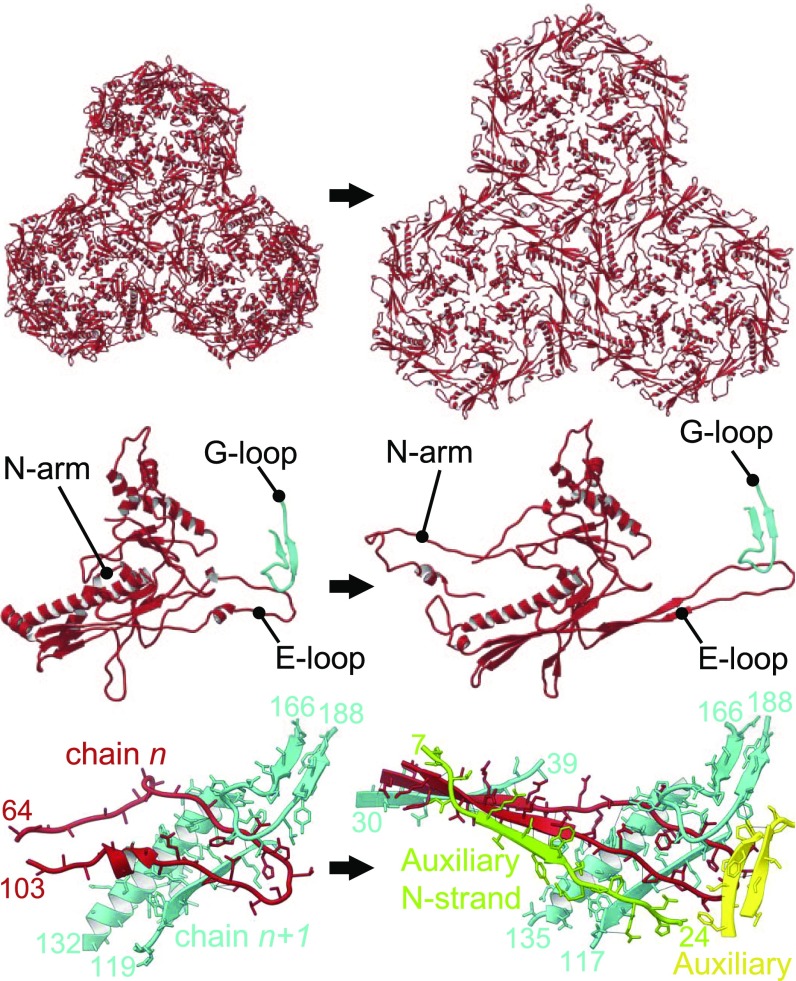
Conformational changes accompanying capsid expansion. (*Left*) The procapsid and (*Right*) the expanded capsid. (*Top*) Three hexons for each expansion state, shown as ribbon diagrams. (*Middle*) Capsid protein monomers along with the G loop from the neighboring subunit of the hexamer, forming the G-loop/E-loop trap. (*Bottom*) Interactions of the E loop with adjacent subunits. E loop is in red, while segments of adjacent capsid protein subunit (Spine helix, P-domain strand, and G loop) are in cyan. In the expanded capsid, segments of two auxiliary protein subunits are shown in green and yellow.

The P23-45 capsomer–capsomer interactions are multilayered (Figs. 3 and 4). At the lowest, internal layer of the capsid, the T loop, N arm, and P domain interact at the threefold and twofold icosahedral and local symmetry axes (Fig. 3*A*). The T loop consists of ∼14 amino acids and extends from the P domain at the local and icosahedral twofold axes, and interdigitates with the P domain of an apposing subunit. The extended N arm reaches toward an adjacent capsomer, forming a “crook” in the expanded capsid, and contributing one strand to a four-stranded β-sheet (Figs. 2*B* and 4). This crook also interacts with the S loop and an apposed crook at the icosahedral and local twofold axes positions. Moving upward through the capsid wall, two further antiparallel strands of the β-sheet are contributed by the E loop that overlays the P domain (Fig. 3*B*). The outermost strand of the sheet is contributed by the N-terminal region of the auxiliary protein (Figs. 3*C* and 4). This interaction is observed only in the expanded capsid, as the auxiliary proteins are only present in the expanded capsid, at the local and icosahedral threefold axes (Figs. 1*D* and 3*D*). Auxiliary proteins of this type were first characterized for bacteriophage λ (25, 26). In the crystal structure of the auxiliary protein from phage P74-26 (32), a close homolog of P23-45, the N-terminal segment, residues 1 to 16, is disordered (Fig. 3*E*), as in the crystal structure of phage λ gpD (25). In contrast, in the context of the expanded P23-45 capsid, the N-terminal segment of the auxiliary protein adopts a well-defined conformation, forming main-chain hydrogen bonds with the E loop of the capsid protein. In the procapsid, the E loop has a relaxed conformation, with its middle section being partially disordered (Fig. 4 and Movies S3 and S4) but with its end locked in a G-loop/E-loop “trap” (Fig. 4), whereby the tip of the E loop is pinned beneath the G loop of a neighboring subunit. Notably, such a G-loop/E-loop interaction is observed in both the procapsid and the expanded capsid states. The G loops complete the outermost layer of the capsid wall and buttress the auxiliary trimers (Fig. 3*C*). Only in the expanded state do the E loops become stretched, adopting a well-defined β-strand conformation and creating the binding site for the N-terminal strand of the auxiliary protein (Figs. 3 and 4). Other significant differences between the expanded and procapsid states are in the N-terminal and C segments of the capsid protein, residues 1 to 23 and 398 to 409, which engage in subunit interactions in the expanded state but are disordered in the procapsid. Notably, the unusual PPFPP motif at the C terminus, residues 401 to 405 (Fig. 2*B*, top left), reinforces subunit interactions via a phenylalanine−tyrosine “lock” between Y226 and F403 of the neighboring C arm (Fig. 2*B*, top right).

### Capsid Volume.

A feature of many T=7 HK97-fold bacteriophages is a skewed dimer-of-trimers arrangement of subunits within procapsid hexons, which adopt a near-sixfold symmetrical arrangement upon capsid expansion. There was no such skew observed in the hexons of P23-45 procapsids, although the ratio of its internal volume versus the expanded capsid, of 0.49, is consistent with observations for other T=7 bacteriophages (Table 1). Strikingly, the P23-45 genome is twice as large as in other T=7 phage. Bacteriophage SIO-2, which has the canonical HK97 capsid fold, but a genome size similar to that of P23-45, instead utilizes a larger (T=12) capsid (33).

### Crystal Structure of the Portal Protein.

**Fig. 5.**
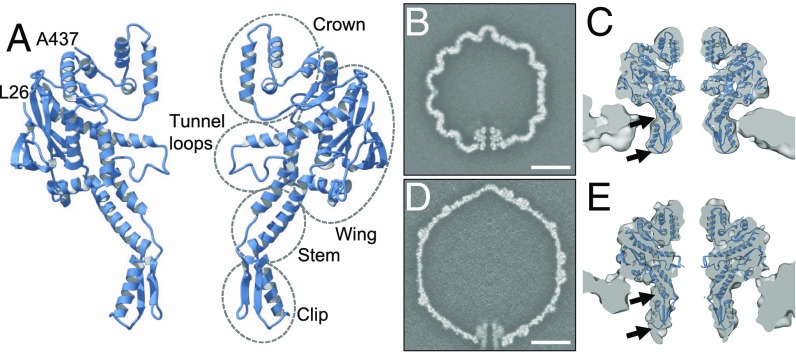
Structures of the portal protein and the unique portal-containing capsid vertex. (*A*) Ribbon diagram of the G20c portal protein crystal structure with annotated domains. Only two apposing subunits are shown, with first (26) and last (437) residues defined in electron density maps, labeled. (*B*) A central slice through the asymmetric reconstruction of the procapsid, and (*C*) portal tunnel region of this map with fitted portal protein subunits. (*D* and *E*) Same as *B* and *C* but for the expanded C5-symmetrized capsid. (Scale bar in *B* and *D*, 200 nm.) Arrows indicate regions which differ most from the fitted crystal structure in the expanded state compared with the procapsid.

A crystal structure was determined for the portal protein from the closely related bacteriophage G20c, which shares 99.3% sequence identity with the P23-45 portal. As the previously determined structure of this portal protein (23) was for a construct lacking 20 N-terminal amino acids, we determined the structure for the portal protein with an intact N terminus. One goal was to test whether this segment adopts an ordered conformation. This structure was refined at 1.95-Å resolution (Fig. 5*A* and *SI Appendix*, Table S2). With three subunits in the asymmetric unit, the 12-subunit oligomer is generated by the crystallographic fourfold axis. The portal protein forms a canonical 12-subunit oligomer with an overall shape consistent with those of portal proteins of other viruses (34). We refer to its domains as Clip, Stem, Wing, and Crown (Fig. 5*A*). The subunit−subunit interactions of this thermostable protein are enhanced compared with portal proteins of mesophilic bacteriophages SPP1 and T4 (34, 35). There are 39 hydrogen bonds (6.6 per 1,000 Å^2^) and seven salt bridges, compared with 26 hydrogen bonds (4.2 per 1,000 Å^2^) and five salt bridges observed in the SPP1 portal protein, and 22 hydrogen bonds (2.9 per 1,000 Å^2^) and four salt bridges present in the T4 portal protein. The first residue clearly defined in the electron density map of the P23-45 portal, Leu26, is located at the outer surface of the portal, indicating that the 25-residue N-terminal segment occupies space around the outside of the portal protein, facing the surrounding proteins in the crystal lattice. Of the first nine residues of the N terminus, five are positively charged, and thus may mediate electrostatic interactions with the capsid.

### Unique Portal Vertex and Normal Mode Analysis.

Reconstructions of the unique portal-containing vertex were performed for both capsid expansion states (Fig. 5 *B*–*E*), using masked classification and not applying icosahedral symmetry. These reconstructions were calculated at 9.3-Å resolution for the procapsid and 9.6-Å resolution for the expanded capsid (*SI Appendix*, Table S1). The portal protein is resolved in both reconstructions, and, within the procapsid, appears closely similar to the crystal structure (Fig. 5 *B* and *C*). In the expanded capsid, however, the Wing of the portal protein rests close to the capsid wall, and the portal protein appears to be stretched along its central axis, with the Stem/Clip regions being elongated and extended away from the capsid (Fig. 5 *D* and *E*).

To investigate conformational states accessible to the portal protein, we performed normal mode calculations with the crystal structure. The lowest energy mode with 12-fold symmetrical motions exhibited periodic compression–extension of the portal protein along its central axis, characterized by a synchronized up-and-down movement of the Wing and Clip (Movie S5). Such conformational changes are consistent with the observed differences between the portal protein in the procapsid and in the expanded capsid (Fig. 5 *B*–*E*). Taken together, these data indicate that the portal protein can change its conformation, and that there is a potential cross-talk between the Wing and Clip; these domains of the portal protein are respectively inside and outside the capsid.

### In Vitro DNA Packaging.

**Fig. 6.**
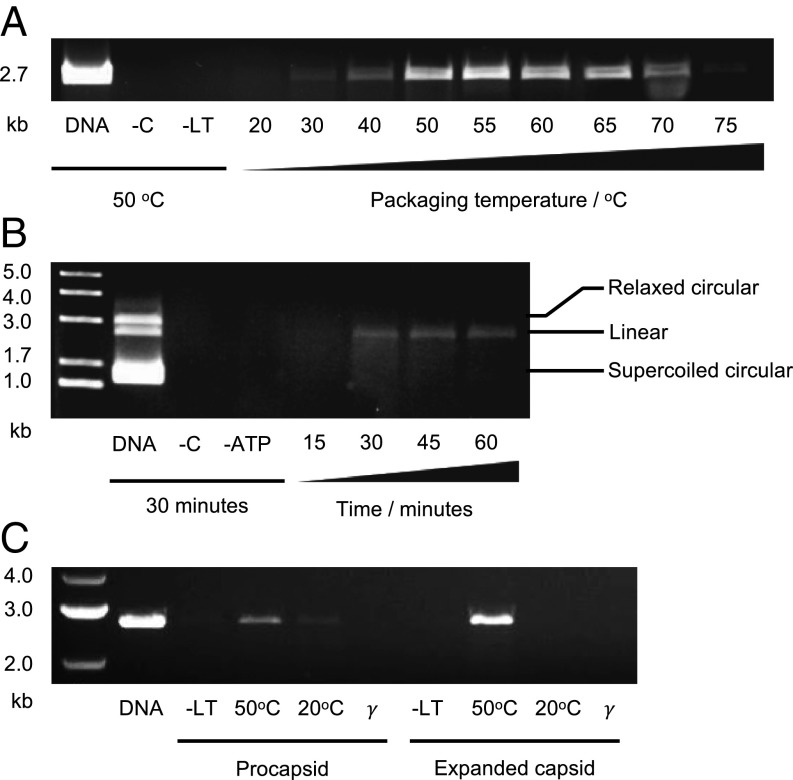
In vitro DNA packaging. (*A*) Comparison of packaging at different temperatures. Lanes from left to right: input DNA (no DNase), negative control with no added capsids, negative control with no added large terminase, packaging reactions at different temperatures. (*B*) Packaging time course. Lanes from left to right: size marker, input DNA (no DNase), negative control with no added capsids, negative control with no added ATP, and packaging for 15, 30, 45, and 60 min. (*C*) Comparison of the ability of the procapsid and expanded capsid to protect DNA from DNase digestion. Lanes from left to right: size marker, input DNA (no DNase), and packaging reactions with no added large terminase (control), with added large terminase at 50 °C and 20 °C, and with ATP substituted by ATPγS (γ).

An in vitro DNA packaging assay was established for empty P23-45 capsids. Isolated empty capsids were tested for their ability to protect plasmid DNA (pUC18) from DNase I digestion in the presence of components required for DNA packaging (Fig. 6). Packaging activity was minimal at 20 °C but increased with rising temperature until ∼50 °C. Optimal activity was observed in the temperature range of 50 °C to 65 °C. At higher temperatures (70 °C to 75 °C), packaging activity decreased (Fig. 6*A* and *SI Appendix*, Fig. S6). No packaged DNA was detected after 15 min of packaging at 50 °C. The intensity of the DNA band after 30 min of packaging was similar to the intensity of bands after 45 and 60 min of packaging. To test the selectivity of the packaging motor, the substrate contained a mixture of linearized, circular supercoiled, and circular relaxed plasmid DNA. Only the linear double-stranded DNA was protected from DNase digestion (Fig. 6*B*), and was therefore packaged. Further experiments compared packaging activity of the procapsid and the expanded capsid. Homogeneity of procapsid samples was monitored by negative staining EM (*SI Appendix*, Fig. S3), and, with mass spectrometry, confirming the absence of auxiliary protein in procapsids and its presence in expanded capsids (*SI Appendix*, Fig. S4). The packaging activity of procapsids was low at 50 °C, with minimal activity observed at 20 °C (Fig. 6*C*). Expanded capsids exhibited high packaging activity at 50 °C, but had no detectable activity at 20 °C. Control experiments carried out at 50 °C in which the large terminase was excluded, or where ATP was substituted with ATPγS, detected no packaging activity. Assessment of procapsids after 30 min of packaging at 50 °C by negative stain EM did not indicate capsid disassembly or expansion (*SI Appendix*, Fig. S5).

## Discussion

### How Can Capsid Size Be Increased Without Changing the T Number?

Virus evolution generally involves the acquisition of genes coding for additional functions, which results in an enlargement of the genome. If the packaging capacity limit of the capsid has been reached, further space for accommodating a larger genome can be achieved in several ways. One way is to adopt a larger T number (that is, use a larger number of capsid protein monomers), with only minor adjustment to their fold. This has occurred throughout evolution, and is demonstrated by the different T numbers of HK97-fold capsids and capsid-like compartments, from the smallest T=1 and T=3 encapsulins (36, 37), to the T=16 herpesviruses (38) and T=52 jumbo bacteriophages (39). Another strategy is to pack the DNA more densely, and, in the herpesviruses (T=16), this appears to have occurred with cytomegalovirus, which has a ∼225-kb genome, compared with the ∼155-kb genome of the herpes simplex virus (40). A third option is capsid expansion, whereby a relatively small procapsid is converted to a larger mature capsid, thereby increasing the storage volume for DNA. These options appear to have an upper limit of DNA packaging densities, which is considered to be near-crystalline in most tailed bacteriophages (4, 5); thus, for many T=7 capsids, there is little potential for packaging additional DNA. A fourth possibility, previously unobserved to our knowledge, is to retain the T number but to increase the capsid volume by increasing the size of the capsomers. This is the strategy employed by bacteriophage P23-45, whereby the capsid lattice constant is increased by ∼3 nm. This large change is accomplished by expansion of the A domain and extension of the E loop relative to the canonical HK97 fold. The auxiliary protein trimers, present in the expanded capsid, further stabilize this state. The N-terminal segment of the auxiliary protein, residues 1 to 16, adopts an ordered conformation only upon incorporation into the (expanded) capsid, and is disordered in the crystal structure (32). Similar observations have been made for the N-terminal segment, residues 1 to 14, of bacteriophage λ auxiliary protein gpD (26). The larger capsid lattice doubles the capsid volume, enabling packaging of twice as large a genome into a T=7 capsid (Table 1). Interestingly, the size of the P23-45 capsid approximates that of SIO-2, a marine bacteriophage with T=12 capsid, which accommodates a genome of 81.2 kb (33). There appears to be a preference in nature for T=7 over T=12 capsids (33, 41), and the advantage of T=7 over T=12 quasi-symmetry may become more significant in the high-temperature environment in which this class of phage propagates. However, the existence of supersized capsid architecture among mesophilic phages cannot be ruled out.

### Can DNA Be Packaged into both Capsid Expansion States?

In addition to its enlarged capsid lattice spacing, the fold of the P23-45 capsid protein must be capable of maintaining capsid integrity at high temperatures (∼70 °C) and in the presence of high internal pressures exerted by packaged DNA. DNA protection assays show that the procapsid and expanded capsids are both capable of initiating packaging with DNA when large terminase is added, and that the in vitro reaction is most efficient at 50 °C to 65 °C. The observation that both procapsid and expanded capsid can package DNA in vitro is consistent with observations for other systems (17, 42–44).

P23-45 expanded capsids exhibit enhanced DNase protection compared with the procapsids. In common, early observations on bacteriophage λ by thin section EM identified expanded capsids with a “grizzled” appearance (45), indicating partial packaging of DNA. Later studies, including those on bacteriophage T4, demonstrated that packaging is not necessarily coupled to expansion (43), and that expanded capsids even display enhanced packaging activity over unexpanded capsids (43, 44). The ability of expanded capsids to package DNA has been further confirmed by single-molecule experiments (17, 42) and structural data (18). Our observations on P23-45 indicate that both capsid expansion states are packaging-competent, but the expanded capsid may be better able to protect DNA either due to greater stability of the capsid or due to enhanced packaging activity which would likely originate from the conformation of the portal.

### How Does the Capsid Influence Portal Protein Conformation?

The portal protein is crucial for the assembly of infectious virions: It assists in scaffold-mediated assembly of the procapsid (46) and serves as the docking site for the DNA packaging motor (18), as a gatekeeper for DNA exit (47), and as the tail attachment site (48). The portal protein’s multifunctionality probably reflects its ability to adopt different conformations. It has been suggested that the P22 portal protein adopts a pseudo-fivefold symmetry to facilitate interaction with the large terminase (49), and a similar mismatch with the capsid vertex must also be satisfied (50). It is therefore not unexpected that our reconstructions of the P23-45 portal vertex indicate differences between the portal protein conformation in the procapsid and expanded capsid (Fig. 5 *B*–*E*), which may result in their differential packaging activities. Likewise, normal mode analysis shows that the lowest-frequency mode with 12-fold symmetry corresponds to a synchronized movement of the Wing and Clip, so that the portal protein oscillates between stretched and compressed conformations (Movie S5). Our analysis of the P23-45 genome termini in sequencing data indicated that this class of viruses encodes a *pac* site in its genomes and uses a headful packaging mechanism (*SI Appendix*, Fig. S7). The portal protein from another *pac* phage, T4 (35), exhibits a similar low-frequency mode (Movie S6), further reinforcing the notion that the portal protein can adopt different conformations, which depend on the stage of virus assembly. Two conclusions can be drawn from these observations. First, the capsid alone can affect the portal protein’s conformation, while the ability of both the procapsid and expanded capsid to package DNA indicates that conformations of the portal protein required for packaging are accessible in both expansion states of the capsid. Second, synchronized movements of the Wing and Clip may serve as a cross-talk mechanism between the two portal domains, suggesting a mechanism by which structural events inside the capsid can be communicated to factors outside. In this way, the capsid could (*i*) present the portal Clip in a compatible conformation to interact with external factors, such as the large terminase, and (*ii*) convey a signal at the termination of packaging.

Future work can exploit the enhanced stability of the thermophage system for eliciting high-resolution structural data for different states of the DNA packaging motor. Single-molecule experiments would complement structural studies, potentially benefiting from reduced packaging rate at ambient temperatures.

## Materials and Methods

Bacteriophage P23-45 particles, and the portal and large terminase proteins, were produced and purified (*SI Appendix*, *SI Materials and Methods*). Cryo-EM structures of capsids as well as the X-ray structure of the portal protein were determined as described in *SI Appendix*, *SI Materials and Methods*. Details of the normal mode analysis, in vitro DNA packaging assays, the genome termini analysis, mass spectrometry of capsids, and densitometric analysis of agarose gels are provided in *SI Appendix*, *SI Materials and Methods*.

The EMDataBank (www.emdatabank.org) deposit of cryo-EM reconstructions includes icosahedral reconstructions of the P23-45 expanded capsid (accession no. EMD-4433) and procapsid (accession no. EMD-4447), asymmetric reconstruction of the procapsid (accession no. EMD-4445), and C5 reconstruction of the expanded capsid (accession no. EMD-4446). The PDB (www.rcsb.org) deposit includes atomic coordinates for the P23-45 expanded capsid (PDB ID code 6I9E) and procapsid (PDB ID code 6IBC), and the G20c portal protein coordinates and structure factors (PDB ID code 6IBG).

## Supplementary Material

Supplementary File

Supplementary File

Supplementary File

Supplementary File

Supplementary File

Supplementary File

Supplementary File
