# Supplementary material for: Cryo-EM structure and in vitro DNA packaging of a thermophilic virus with supersized T=7 capsids
Source: Proc Natl Acad Sci U S A. 2019 Feb 8;116(9):3556–61. doi: 10.1073/pnas.1813204116 (PMC6397560; doi:10.1073/pnas.1813204116)
Supplement: Supplementary File [file pnas.1813204116.sapp.pdf]

Cryo-EM structure and in vitro DNA packaging of a thermophilic virus with supersized T=7 capsids

Oliver W. Bayfield[a,b], Evgeny Klimuk[c,d], Dennis C. Winkler[b], Emma L. Hesketh[e], Maria Chechik[a], Naiqian Cheng[b], Eric C. Dykeman[f], Leonid Minakhin[g], Neil A. Ranson[e], Konstantin Severinov[c,d,g], Alasdair C. Steven[b], Alfred A. Antson[a]

[a] York Structural Biology Laboratory, Department of Chemistry, University of York, York, YO10 5DD, United Kingdom.

[b] Laboratory of Structural Biology Research, National Institute of Arthritis Musculoskeletal and Skin Diseases, NIH, Bethesda, MD 20892, USA.

[c] Center for Life Sciences, Skolkovo Institute of Science and Technology, Skolkovo 143025, Russia.

[d] Institute of Molecular Genetics, Russian Academy of Sciences, Moscow 123182, Russia.

[e] Astbury Centre for Structural Molecular Biology, University of Leeds, Mount Preston Street, Leeds, LS2 9JT, United Kingdom.

[f] Department of Mathematics, University of York, York, YO10 5DD, United Kingdom.

[g] Waksman Institute for Microbiology, Rutgers, The State University of New Jersey, NJ 08854, USA.

Supporting Information

SI Materials and Methods

Bacteriophage propagation and capsid isolation

A 400 ml volume of TB medium (0.8 % w/v Tryptone, 0.4 % w/v NaCl, 0.2 % w/v Yeast extract) was innoculated with 15 ml of an overnight (~16 hour) culture of *Thermus thermophilus* HB8 and grown for approximately 3 hours at 70 ^{o}C and 200 rpm, until and A_{600} of 0.2 had been reached. The culture was infected with P23-45 by addition of 400 μl of a $2x10^{9}$ pfu/ml stock, and after a further 2 hours the culture had fully lysed, with an A_{600} of 0.05. The lysate was moved to 4 ^{o}C and 1 μl of Benzonase was added, and adjusted to 0.3 mM EDTA. Cell debris was removed by centrifugation at 6000 x g at 4 ^{o}C for 30 minutes. Virus particles were concentrated by ultracentrifugation at 140000 x g at 4 ^{o}C for 2 hours. Pellets were resuspended in 200 μl of SM buffer containing 50 mM Tris-HCl pH 7.5, 100 mM NaCl, 10 mM $MgSO_4$, 0.5 mM $CaCl_2$. Loaded onto a stepped CsCl gradient at densities of 1.25, 1.3, 1.4 and 1.55 g/ml, containing 20 mM Tris-HCl pH 7.5, 10 mM $MgSO_4$, in an Ultra-Clear SW28 tube (Beckman Coulter). Gradients were centrifuged at 140000 x g at 8 ^{o}C for 3 hours. For finer separation an isopycnic gradient was established by adjusting the density of the lysate to 1.4 g/ml with a saturated CsCl solution and centrifuging at 110000 x g at 10 ^{o}C for 40 hours in an SW28 rotor. Bands were extracted by side puncture and dialysed against 20 mM Tris-HCl pH 8.0, 50 mM NaCl, 10 mM $MgCl_2$ at 4 ^{o}C for 18 hours. Further separation could be achieved through a 10–30 % v/v glycerol gradient containing 50 mM NaCl, 20 mM Tris-HCl pH 8.0, 10 mM $MgCl_2$, at 130000 x g at 10 ^{o}C for 2 hours. Alternatively, anion exchange chromatography was employed. Capsids were dialysed against 100 mM NaCl, 20 mM Tris-HCl pH 8.0, 10 mM $MgCl_2$ for at 4 ^{o}C for 18 hours. Concentrations were estimated by A_{280} measurements and sample quality assessed by negative staining transmission electron microscopy, SDS-PAGE, and mass spectrometry.

Portal protein purification

The portal protein gene for bacteriophage G20c encoding residues 1–438 was cloned into vector pET22b between NdeI and XhoI restriction sites with a C-terminal His-tag downstream. Protein was expressed in *E.coli* BL21 (DE3) at 16 ^{o}C for 18 hours by induction with 1 mM IPTG. Cells were lysed in 20 mM Imidazole, 50 mM Tris-HCl pH 7.5, 1 M NaCl, 5 % v/v glycerol. The lysate was applied to a 5 ml HisTrap FF column and eluted with a linear gradient to 500 mM Imidazole,

50 mM Tris-HCl pH 7.5, 1 M NaCl, 5 % v/v glycerol over 20 column volumes. Fractions containing the target protein were pooled and loaded onto a Superose 6 10/30 column in 20 mM Tris-HCl pH 7.5, 1 M NaCl, 5 % v/v glycerol. Fractions containing the target protein were pooled and concentrated in a 100 kDa MWCO centrifugal concentrator to 20 mg/ml (39.8 μM).

Large terminase purification

The P23-45 large terminase gp85 construct was fused to an N-terminal SUMO-His$_6$ tag. The gene was codon optimised for expression in *E.coli*. and synthesized by ThermoFisher Scientific, and cloned into vector pET151-DTOPO between TOPO sites, downstream of a T7 promoter and *lac* operator. The construct was overexpressed in *E.coli* BL21 (DE3) at 37 ^{o}C in autoinduction media (1) for 24 hours. Cells were harvested by centrifugation at 7000 x g at 4 ^{o}C for 25 minutes and stored at -80 ^{o}C. Cells were resuspended in buffer containing 30 mM Imidazole, 50 mM Tris-HCl pH 8.0, 200 mM NaCl, 200 mM KCl, 10 mM $MgSO_4$, at 2 ml/g pellet. Benzonase (Sigma) was added at 1.3 μl/100 ml, and lysozyme at 0.1 g/100 ml, and adjusted to 3 mM EDTA. Cells were lysed by sonication on ice, and lysate was clarified by centrifugation at 35000 x g at 4 ^{o}C for 1 hour. The clarified lysate was loaded onto a 5 ml HisTrap FF column and eluted with a linear gradient from 30 mM to 500 mM Imidazole over 20 column volumes. Fractions containing the target protein were pooled and Ulp1 protease was added to cleave the SUMO-His$_6$ tag, hence leaving no additional residues introduced by cloning. The digested sample was purified by size exclusion chromatography using a HiLoad Superdex 200 pg 16/600 column. Fractions containing the target protein were pooled and adjusted to 1 M $(NH_4)_2SO_4$ with 3.9 M $(NH_4)_2SO_4$ pH 7.0, loaded onto a 1 ml HiTrap Phenyl HP column, and eluted with a linear gradient from 1.3 M to 0 M $(NH_4)_2SO_4$ over 20 column volumes. Fractions containing the target protein were pooled and dialysed for 18 hours at 4 ^{o}C into 20 mM Tris-HCl pH 8.0, 150 mM NaCl, 150 mM KCl, 10 mM $MgSO_4$. The purified protein was concentrated to 348 μM and had a A_{260}/A_{280} value of 0.52 and was shown to be exclusively full-length protein without detectable levels of C-terminal degradation by SDS-PAGE. Aliquots were flash frozen in liquid nitrogen for storage at -80 ^{o}C. Concentrations were estimated by A_{280} measurement. Whilst enzyme activity was ultimately confirmed by the DNA packaging assay, initially nuclease activity was confirmed by digestion of pUC18 plasmid DNA visualised by agarose gel electrophoresis, and ATPase activity was confirmed using the EnzCheck phosphate assay kit as per the manufacturer's instructions.

Cryo-electron microscopy and image processing

Quantifoil R1.2/1.3 400 Mesh Copper grids (Agar scientific) were plasma-cleaned using a PELCO easiGLOW at 0.26 mbar, 10 mAmp for 30 seconds. Grids were plunge frozen in liquid ethane using a Vitrobot Mark IV at 90 % humidity at 10 ^{o}C. Grids were imaged on a ThermoFisher Scientific Titan Krios 300 kV field emission TEM (Astbury BioStructure Laboratory, University of Leeds) at a nominal magnification of 75000x and a nominal defocus range of 0.5 to 2.5 μm, with a spherical aberration of 2.7 mm. Data were collected using the EPU software package. Movies were recorded on a Falcon 3EC (ThermoFisher Scientific) in integrating mode over a 2 second exposure composed of 79 frames, with 2 exposures taken per hole. The calibrated pixel size was 1.065 Å. Two data sets of 2706 and 5052 movies combined for processing, and had accumulated doses of 99 and 96 e/Å^2, respectively, to allow for subsequent dose-weighting. Image processing was performed using RELION 2.10 (2). Movie frame alignment was performed with MotionCor2 1.0.2 (3), using patches size of 5 x 5, B-factor of 150, without grouping or binning frames. CTF estimation was performed using CTFFIND4.1 (4) without dose-weighting, and particles were extracted from dose-weighted motion-corrected micrographs. The box size for the procapsid was 972 pixels and for the expanded capsid was 1032 pixels. Particles were downsampled by a factor of 1.5 resulting in a pixel size of 1.5975 Å/pixel. Particles were subsequently 2D classified and subsets were select for 3D reconstructions. An initial reference model was generated from particles with randomised orientations, resulting in a spherical model. Subsequent cycles of refinement used an icosahedral capsid reference low-pass filtered to 60 Å. Refinement was carried out in RELION 2.10 using 3D auto-refine imposing icosahedral symmetry. A total of 38,044 particles contributed to the icosahedral procapsid reconstruction, and 2,372 particles contributed to the icosahedral expanded capsid reconstruction. For the expanded capsid, CTF estimates were additionally refined using CTFRefine in RELION 3.0. Postprocessing was carried out using a soft-edged mask, supplying the MTF file of the detector. The postprocessing resolution of the procapsid was 4.39 Å and for the expanded capsid was 3.74 Å, according to 0.143 FSC criterion (5). Data collection and refinement statistics are summarized in the table below (**Table S1**). Figures were generated in UCSF Chimera (6).

Reconstructions depicting the portal protein

In the icosahedral reconstructions calculated using RELION a faint density of the portal protein was apparent at each vertex. The relion_particle_symmetry_expand function was used to generate 60 icosahedral symmetry-related orientations per particle. Particles were re-extracted with downsampling by a factor of 6 and subjected to 3D classification into 10 classes without changing orientations or imposing symmetry (C1), with a high resolution limit of 12 Å, using a soft-edged mask centered at one vertex. A soft-edged mask was generated using the portal protein crystal structure applying a low-pass filter of 60 Å and expanding the mask edge at a low threshold. After 3D classification, one class contained density for the portal protein. Particles from this class were re-extracted, downsampling by a factor of 1.5 from the original micrographs. Subsequent rounds of 3D classification without changing orientations, with a high resolution limit of 5–8 Å, resolved the symmetry mismatch between the portal and procapsid vertex. In the expanded capsid, C5 symmetry was imposed in the final reconstruction. Where more than one symmetry-related orientation of the same original particle remained, only the particle with the highest LogLikeliContribution value was retained in a new version of the ".star" file. Particles were split into half-sets and reconstructed without changing orientations, using C1 symmetry for the procapsid and imposing C5 symmetry for the expanded capsid. A total of 16758 particles contributed to the C1 procapsid reconstruction, and 902 particles contributed to the C5 expanded capsid reconstruction. Postprocessing was carried out using a soft-edged mask, supplying the MTF file of the detector. The postprocessing resolution for the asymmetric procapsid was 9.33 Å and for the C5 expanded capsid was 9.56 Å, according to the 0.143 FSC criterion (5). Refinement statistics are summarized in **Table S1**.

Capsid structure modelling and analysis

A section of the expanded capsid map was extracted and segmented in UCSF Chimera using Segger (6, 7) for initial modelling. An atomic model was initially built using Phenix Map to Model, with manual rebuilding in Coot (8) followed by refinement using Phenix Real Space Refine (9). The resulting model for the expanded capsid was fitted into the procapsid map and manually adjusted in Coot, followed by refinement with Phenix. Refinement statistics are summarized in the table below (**Table S1**). For internal capsid volume measurement, capsid maps were inverted in EMAN2 (10) and low-pass filtered of 20 Å, and the capsid core was segmented using

Segger and its volume measured in UCSF Chimera. Where a capsid crystal structure was used the PDB was first converted to a volume (.mrc) in UCSF Chimera.

Portal protein crystal structure determination

For crystallization, the sitting-drop vapor diffusion method was used. Protein concentration was 20 mg/ml (39.8 μM). In addition, a 42 residue synthesized peptide (NovoPro), corresponding to the C-terminal segment of the large terminase protein gp85, was added to a final concentration of 2 mg/ml (413 μM) in drops, with the ratios of 100 nl protein : 50 nl peptide : 150 nl reservoir solution. Crystals formed in 40 % v/v MPD, 200 mM NaH_2PO_4. X-ray data were collected at Diamond Light Source, Oxfordshire, UK (**Table S2**). The structure was determined by molecular replacement using the CCP4 suite of programs (11). Molecular replacement was carried out using the coordinates available for a N-terminally truncated version of the portal protein (PDB code 4ZJN). The structure was refined by REFMAC, with manual rebuilding performed in Coot (**Table S2**). Data collection and refinement statistics are summarized in the table below.

Portal protein Normal Mode Analysis

Normal modes were computed using an Elastic Network Model (ENM) where the motions of only the $C\alpha$ carbon atoms of the protein backbone were considered. The atomic coordinates were postprocessed to remove all atoms except for the $C\alpha$ atoms. Normal modes were computed using an ENM with an 8 Å cut-off for the interactions, and the lowest 100 frequency modes were computed. Modes of interest occurred within the 3 lowest frequency modes.

In vitro DNA packaging assay

The following components were incubated: 2 μl of ScaI linearised pUC18 DNA at 250 ng/ul, 17.5 μl of P23-45 empty capsid at 40 nM, 2.5 μl of ATP at 80 μM, 2 μl of large terminase at 10 μM containing 20 mM Tris-HCl pH 8.0, 300 mM NaCl, 10 mM $MgCl_2$. Following incubation, 1 μl of DNase I Amplification grade at 1 unit/μl (Simga-Aldrich) was added to digest unprotected DNA, followed by 2 μl of EDTA pH 8.0 at 0.5 M, 1 μl of Proteinase K PCR Grade at 20 mg/ml (Roche), and 5 μl of gel loading dye. Samples were incubated for 30 minutes at 60 ^{o}C before visualizing by agarose gel electrophoresis.

Bacteriophage Genome Termini Analysis

DNA was isolated from concentrated and purified high titer P23-45 stock by incubation in 0.5 % w/v SDS, 20 mM EDTA and 50 µg/ml proteinase K at 56 °C for 1–3 h. The DNA was extracted with phenol-chloroform and precipitated with ethanol supplemented with sodium acetate (12). Genome sequencing was performed using Miseq Illumina in pair-end 250-bp long reads mode according to manufacturer's protocols (13). The generated reads were analyzed with the PhageTerm software (14) on a Galaxy-based server (https://galaxy.pasteur.fr) with default parameters.

Mass spectrometry of capsids (LC-MS/MS)

Protein in 50 mM ammonium bicarbonate aqueous solution was reduced with 50 mM tris(2-carboxyethyl)phosphine and alkylated with 200 mM methyl methanethiosulfonate. Digestion of protein was achieved by addition of 0.2 g of sequencing-grade modified porcine trypsin (Promega) with incubation at 37 ^{o}C for 16 hours. The peptide mixture was loaded onto a nanoAcquity UPLC system (Waters) equipped with a nanoAcquity Symmetry C18, 5 µm trap (180 µm x 20 mm, Waters) and a nanoAcquity HSS T3 1.8 µm C18 capillary column (75 m x 250 mm, Waters). The trap wash solvent was 0.1 % v/v aqueous formic acid and the trapping flow rate was 10 µl/min. The trap was washed for 5 minutes before switching flow to the capillary column. Separation used a gradient elution of two solvents (solvent A: aqueous 0.1 % v/v formic acid; and solvent B: acetonitrile containing 0.1 % v/v formic acid). The capillary column flow rate was 350 nL/min and the column temperature was 60°C. The gradient profile was linear 2–35 % B, over 20 minutes. All runs then proceeded to wash with 95 % solvent B for 2.5 minutes. The column was returned to initial conditions and re-equilibrated for 25 minutes before subsequent injections. The nanoLC system was interfaced with a maXis HD LC-MS/MS system (Bruker Daltonics) with CaptiveSpray ionisation source (Bruker Daltonics). Positive ESI-MS and MS/MS spectra were acquired using AutoMSMS mode. Instrument control, data acquisition and processing were performed using Compass 1.7 software (microTOF control, Hystar and DataAnalysis, Bruker Daltonics). Instrument settings were: ion spray voltage: 1,450 V, dry gas: 3 L/min, dry gas temperature 150°C, ion acquisition range: m/z 150-2,000, MS spectra rate: 5 Hz, MS/MS spectra rate: 5 Hz at 2,500 cts to 25 Hz at 250,000 cts, cycle time: 1 s, quadrupole low mass: 300 m/z, collision RF: 1,400 Vpp, transfer time 120 ms. The collision energy and isolation width settings were automatically calculated using the AutoMSMS fragmentation table,

absolute threshold 200 counts, preferred charge states: 2–4, singly charged ions excluded. A single MS/MS spectrum was acquired for each precursor and former target ions were excluded for 0.8 minutes unless the precursor intensity increased fourfold. Tandem mass spectra were searched against the unrestricted UniProt database concatenated with an in-house database containing expected sequences (total sequences = 557095) using a locally-running copy of the Mascot program (Matrix Science Ltd., version 2.6), through the Mascot Daemon interface (version 2.6). Search criteria specified: Enzyme, trypsin; Fixed modifications, Methylthio (C); Variable modifications, Oxidation (M); Peptide tolerance, 10 ppm; MS/MS tolerance, 0.1 Da; Instrument, ESI-QUAD-TOF. Results were filtered to achieve a false discovery rate of <1 % as assessed by searching using the same parameters against a reversed database. Molar percentages (Mol%) were calculated from Mascot emPAI values by expressing individual values as a percentage of the sum of all emPAI values in the sample (15).

Densitometric analysis of agarose gels

Gel images were analyzed using ImageJ software (imagej.nih.gov/ij/). Regions containing bands were boxed in separate lanes using the rectangular selection tool, and analyzed using the ‘Plot lanes’ tool. Areas under the curve were measured and normalized against the input DNA (control) lane.

SI Tables

Table S1. Cryo-EM Data Collection and Refinement Statistics.

	Procapsid, Icosahedral	Expanded capsid, Icosahedral	Procapsid, C1	Expanded capsid, C5
**Data collection**				
Microscope	Titan Krios			
High Tension / kV	300			
Pixel size / Å	1.065			
Spherical aberration / mm	2.7			
Nominal magnification	75000			
Nominal defocus / μm	0.5–2.5			
Detector (mode)	Falcon 3EC (integrating)			
Dose, pre-weighted / eÅ^{-2}	99			
**Refinement and model statistics**				
Symmetry	I	I	C1	C5
Resolution (FSC 0.143)	4.39	3.74	9.33	9.56
Map-to-model correlation	0.743	0.822	-	-
MolProbity score	1.99	1.77	-	-
EMRinger score	0.77	3.10	-	-
RMS deviations			-	-
Bond lengths / Å	0.007	0.009	-	-
Bond angles / $^{\circ}$	1.1	0.9	-	-
Ramachandran plot / %			-	-
Favored	91.1	91.1	-	-
Allowed	8.7	8.8	-	-
Outliers	0.2	0.1	-	-

Table S2. X-ray Data Collection and Refinement Statistics.

Data collection		Refinement and model statistics	
X-ray source	I04-1, DLS	Resolution range / Å	94.2 – 1.95 (2.00 – 1.95)
Wavelength / Å	0.92819	*R*-factor / %	15.9 (35.4)
		R-free / %	21.6 (41.9)
Resolution range / Å (outer shell)	94.2 – 1.95 (1.98 – 1.95)	Reflections working	104315 (7662)
		Reflections free	1013 (52)
Space group	I4	Number of protein atoms	10346
		Number of solvent molecules	611
Unit-cell / Å	*a* = 159.1		
Number of unique reflections	105514 (7752)	RMS deviations	
		Bond lengths / Å	0.006
Completeness / %	99.8 (99.9)	Bond angles / °	1.4
Redundancy	6.8 (7.0)		
$I/\sigma(I)$	6.2 (0.6)	Ramachandran plot / %	
R_{merge} / %	10.3 (119.9)	Favoured	97.6
$CC_{1/2}$ / %	99.9 (62.6)	Allowed	2.4
		Outliers	0.0

Values in parentheses correspond to the highest resolution shell.

SI Figures

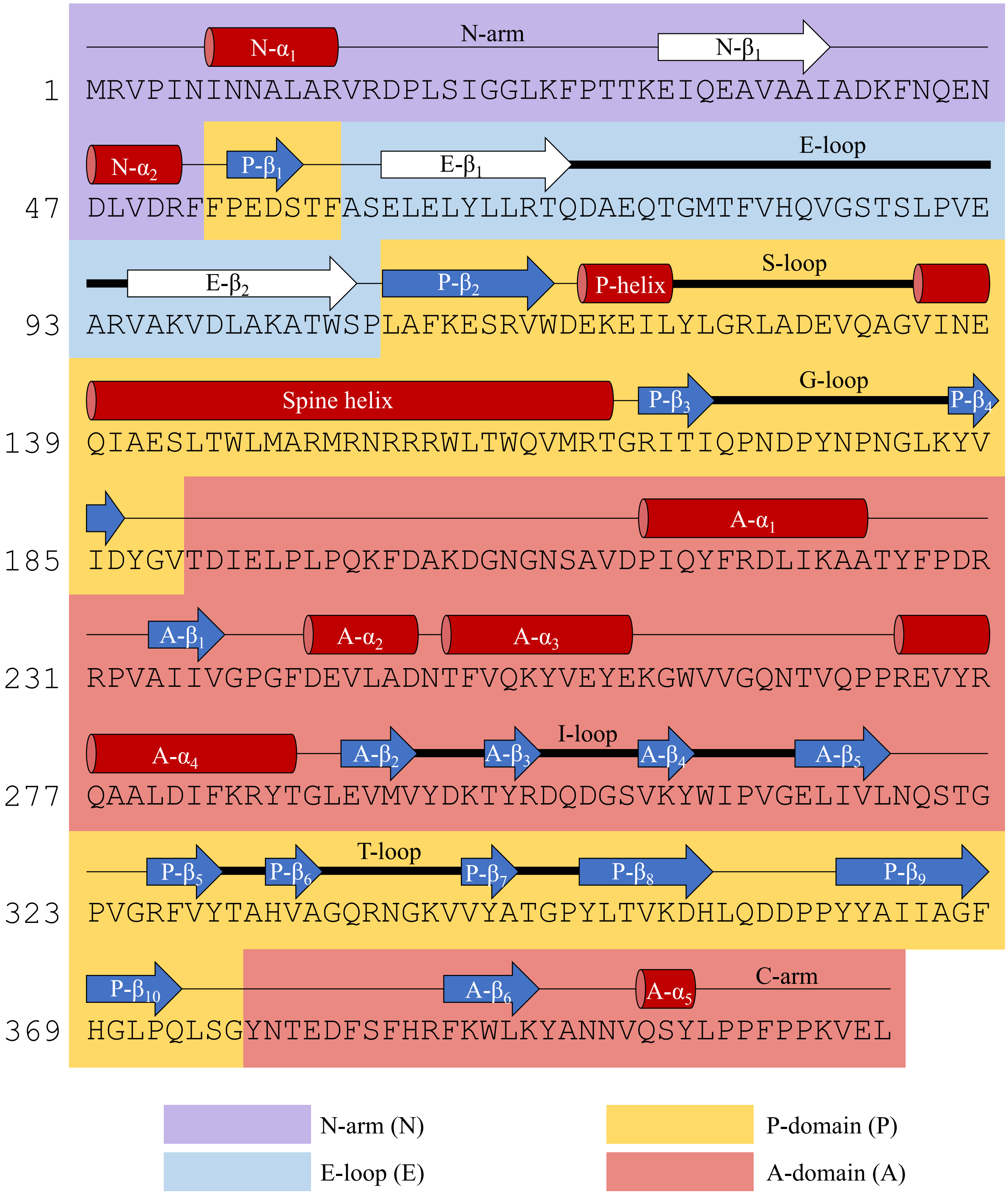

Figure S1. P23-45 capsid protein gp89 sequence annotated with secondary structure elements and domains identified for the expanded capsid. β-strands involved in the intersubunit β-sheet formed between the capsid and auxiliary protein are in white.

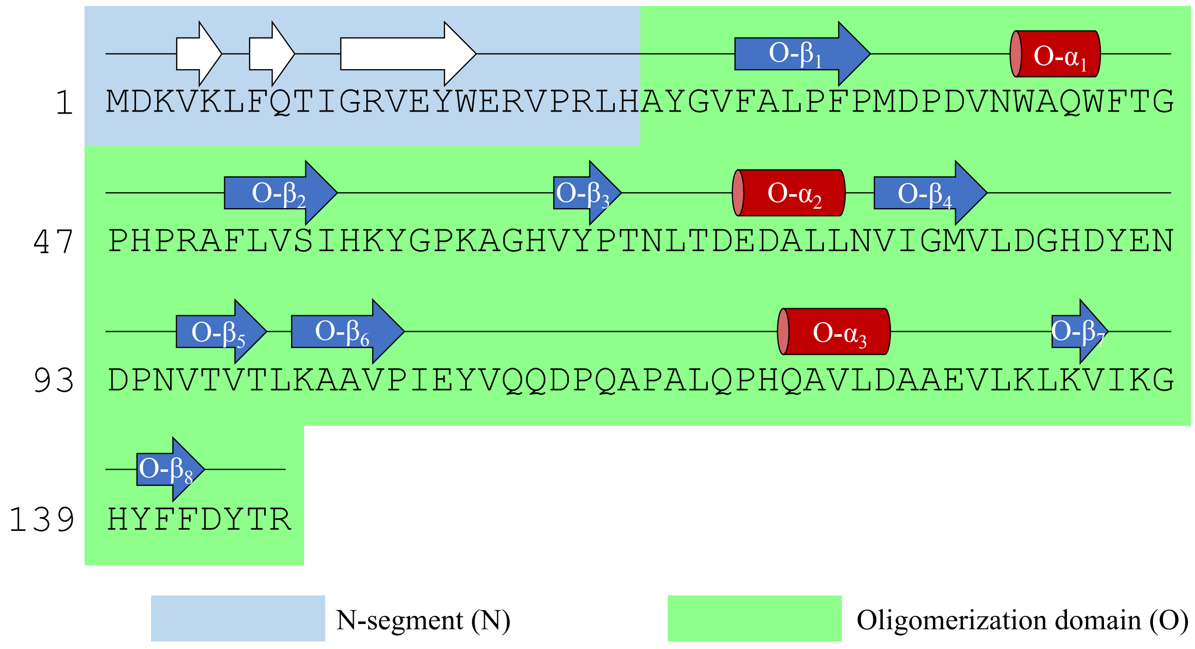

Figure S2. P23-45 auxiliary protein gp88 sequence annotated with secondary structure elements and domains. β-strands contributed to the intersubunit β-sheet formed between the capsid and auxiliary protein are in white.

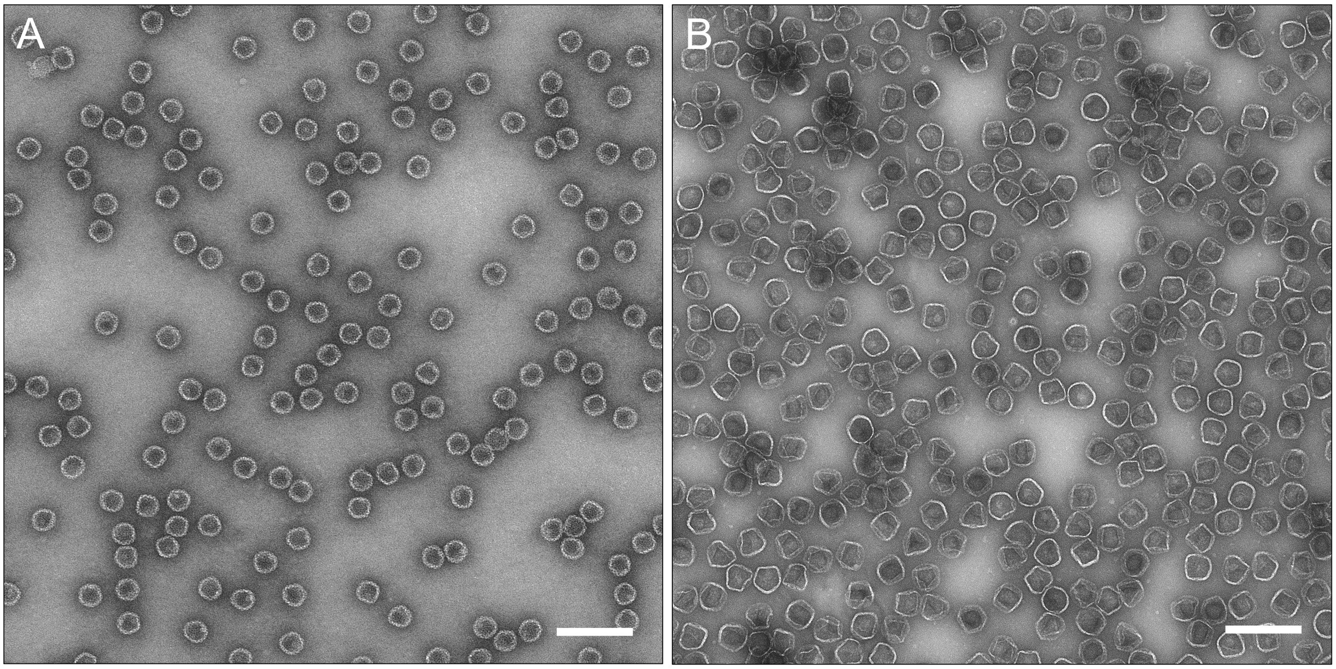

Figure S3. Electron micrographs of purified capsids used for *in vitro* packaging assays. (A) procapsids, (B) empty expanded capsids. Samples were stained with 2% w/v uranyl acetate. Scale bars are 200 nm.

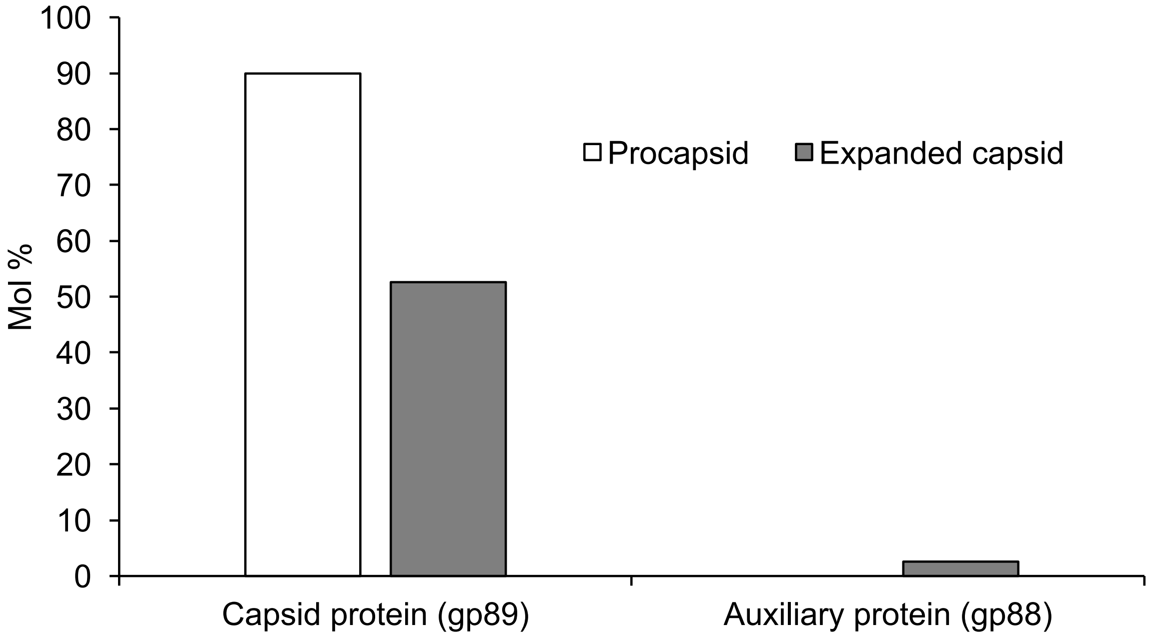

Figure S4. Molar percentages of the capsid and auxiliary proteins detected by mass spectrometry in purified samples of procapsids and expanded capsid.

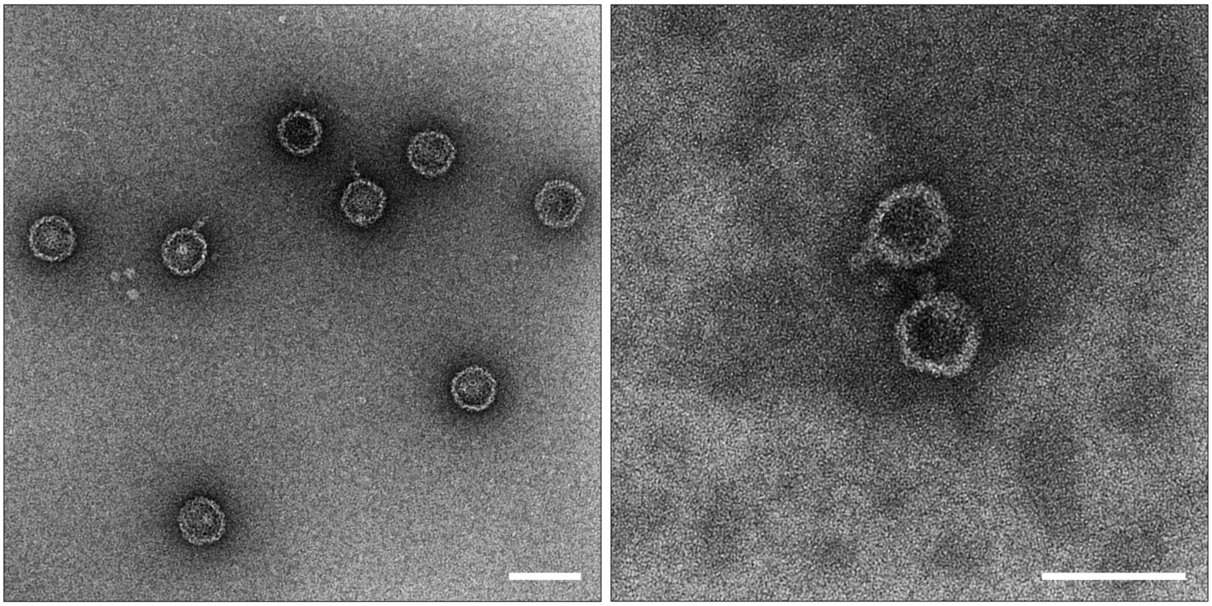

Figure S5. Representative electron micrographs of packaging reaction mixture for procapsids. Procapsids were stained with 2 % w/v uranyl acetate, after packaging with plasmid DNA at 50 ^{o}C for 30 minutes. Scale bar is 100 nm.

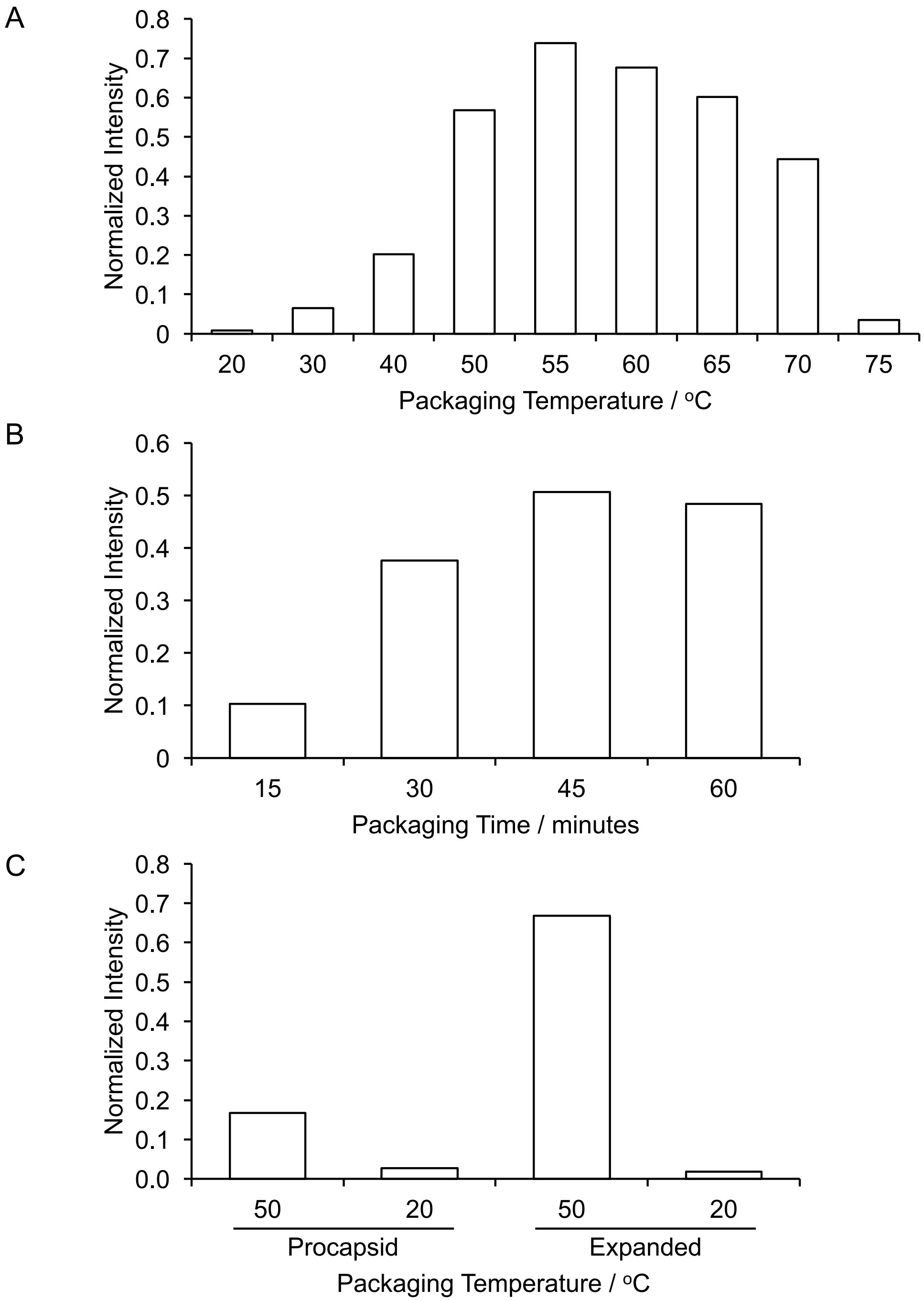

Figure S6. *In vitro* DNA packaging. Densitometric analysis of agarose gels shown on Figure 6. Band intensities were normalized to the input (control lane) DNA density.

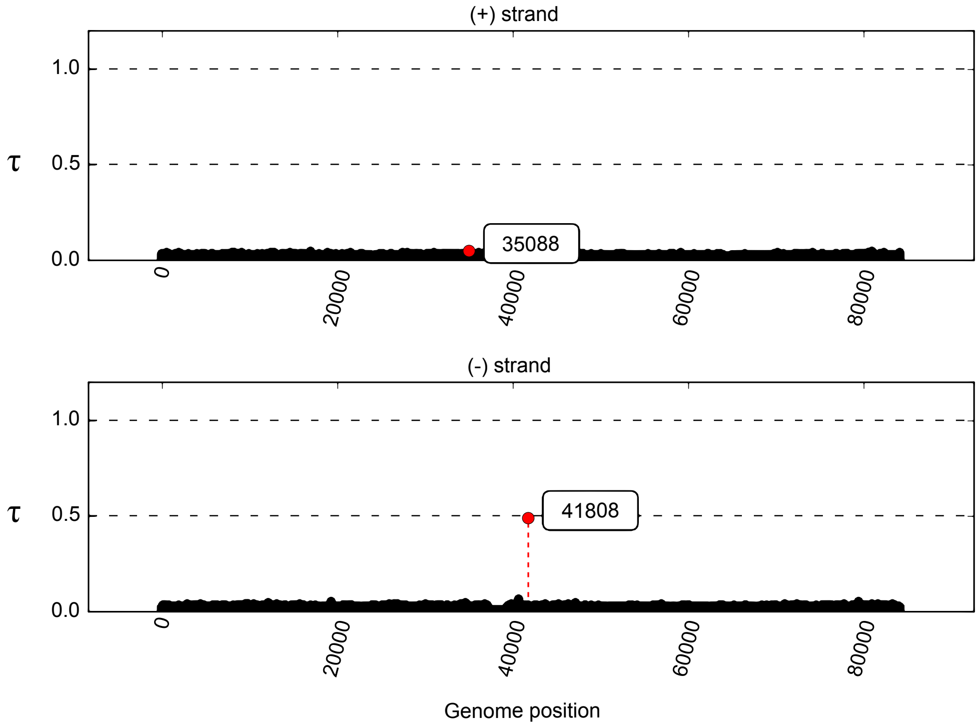

Genome	Ends	Left	Right	Class
84201 (NC_009803.1)	Redundant	Distributed	41808	Headful (pac)

Figure S7. PhageTerm Analysis of P23-45 genome sequencing data. PhageTerm τ value is plotted against genome position (bp), for positive (+) and negative (-) strands.

SI Movies

Movie S1. Icosahedral reconstruction of the procapsid. Rotation is around the 5-fold axis.

Movie S2. Icosahedral reconstruction of the expanded capsid. Rotation is around the 5-fold axis.

Movie S3. Morph between the procapsid (PDB 6IBC) and expanded capsid (PDB 6I9E) states. Three hexons of the major capsid protein gp89 are shown.

Movie S4. Morph between the procapsid (PDB 6IBC) and expanded capsid (PDB 6I9E) states. Movie generated for a single subunit.

Movie S5. Low frequency mode of the G20c portal protein. Normal mode analysis was performed using the PDB file 6IBG.

Movie S6. Low frequency mode of the bacteriophage T4 portal protein. Normal mode analysis was performed for the PDB structure 3JA7.

SI References

1. Studier FW (2005) Protein production by auto-induction in high-density shaking cultures. *Protein Expr Purif* 41(1):207–234.
2. Scheres SHW (2012) RELION: Implementation of a Bayesian approach to cryo-EM structure determination. *J Struct Biol* 180(3):519–530.
3. Zheng SQ, et al. (2017) MotionCor2 - anisotropic correction of beam-induced motion for improved cryo-electron microscopy. *Nat Methods* 14(4):331–332.
4. Rohou A, Grigorieff N (2015) CTFFIND4: Fast and accurate defocus estimation from electron micrographs. *J Struct Biol* 192:216–221.
5. Rosenthal PB, Henderson R (2003) Optimal determination of particle orientation, absolute hand, and contrast loss in single-particle electron cryomicroscopy. *J Mol Biol* 333(4):721–745.
6. Pettersen EF, et al. (2004) UCSF Chimera - A visualization system for exploratory research and analysis. *J Comput Chem* 25(13):1605–1612.
7. Pintiliea GD, Zhangb J, Goddardc TD, Chiu W, Gossardd DC (2010) Quantitative analysis of cryo-EM density map segmentation by watershed and scale-space filtering, and fitting of structures by alignment to regions. *J Struct Biol* 170(3):427–438.
8. Emsley P, Lohkamp B, Scott WG, Cowtan K (2010) Features and development of Coot. *Acta Crystallogr Sect D Biol Crystallogr* 66(4):486–501.
9. Adams PD, et al. (2010) PHENIX: A comprehensive Python-based system for macromolecular structure solution. *Acta Crystallogr Sect D Biol Crystallogr* 66(2):213–221.
10. Tang G, et al. (2007) EMAN2: An extensible image processing suite for electron microscopy. *J Struct Biol* 157(1):38–46.
11. Winn MD, et al. (2011) Overview of the CCP4 suite and current developments. *Acta Crystallogr D Biol Crystallogr* 67(Pt 4):235–42.
12. Green MR, Sambrook J (2012) *Molecular cloning: A Laboratory Manual* (Cold Spring Harbor Laboratory Press, New York). Fourth doi:10.1016/B978-0-12-418687-3.00007-0.
13. Musharova O, et al. (2016) Spacer-length DNA intermediates are associated with Cas1 in cells undergoing primed CRISPR adaptation. *Nucleic Acids Res* 45(6):3297–3307.
14. Garneau JR, Depardieu F, Fortier LC, Bikard D, Monot M (2017) PhageTerm: A tool for fast and accurate determination of phage termini and packaging mechanism using next-generation sequencing data. *Sci Rep* 7(1):1–10.
15. Ishihama Y, et al. (2005) Exponentially Modified Protein Abundance Index (emPAI) for Estimation of Absolute Protein Amount in Proteomics by the Number of Sequenced Peptides per Protein. *Mol Cell Proteomics* 4(9):1265–1272.
